# Physicians perceived usefulness of high-cost diagnostic imaging studies: results of a referral study in a German medical quality network

**DOI:** 10.1186/1471-2296-6-22

**Published:** 2005-06-07

**Authors:** Antonius Schneider, Thomas Rosemann, Michel Wensing, Joachim Szecsenyi

**Affiliations:** 1 Department of General Practice and Health Services Research, University of Heidelberg

## Abstract

**Background:**

Medical and technological progress has led to increased numbers of diagnostic tests, some of them inducing high financial costs. In Germany, high-cost diagnostic imaging is performed by a medical specialist after referral by a general practitioner (GP) or specialist in primary care. The aim of this study was to evaluate the physicians' perceived usefulness of high-cost diagnostic imaging in patients with different clinical conditions.

**Methods:**

Thirty-four GPs, one neurologist and one orthopaedic specialist in ambulatory care from a Medical Quality Network documented 234 referrals concerning 97 MRIs, 96 CTs-scan and 41 intracardiac catheters in a three month period. After having received the test results, they indicated if these were useful for diagnosis and treatment of the patient.

**Results:**

The physicians' perceived usefulness of tests was lowest in suspected cerebral disease (40% of test results were seen as useful), cervical spine problems (64%) and unexplained abdominal complaints (67%). The perceived usefulness was highest in musculoskeletal symptoms (94%) and second best in cardiological diseases (82%).

**Conclusion:**

The perceived usefulness of high-cost diagnostic imaging was lower in unexplained complaints than in specific diseases. Interventions to improve the effectiveness and efficiency of test ordering should focus on clinical decision making in conditions where GPs perceived low usefulness.

## Background

The continuous medical and technological progress has led to a rising use of high tech diagnostic tests which are often expensive. For that reason many efforts have been undertaken to enhance the effectiveness and efficiency of referrals for diagnostic tests. Studies have pointed out a wide range of reasons for referrals including patients demand for extensive diagnostics. For instance, referral patterns were related to the physicians' attitudes to their role [1] and to the interaction between the physician and patient [2]. Also the social context seems to have a high influence for referral for further diagnostics [3]. Nevertheless, the variation of referral rates remains largely unexplained [4].

Concerning expensive diagnostic procedures, Robling et al. found that the referral for diagnosis with MRI had biomedical, personal and contextual reasons [5]. Particularly "vague complaints" were related to a high likelihood for test ordering. A large observational, cross-sectional study revealed the influence of patients' expectations about test ordering for further diagnostic to clarify vague symptoms [6]. As the variation of referrals seems to be related with high expenditures of health care systems, health policy makers are seeking improvement in this area [7].

In general, there is no formal gatekeeping role for the GP in the German health care system and ambulatory care comprises almost all specialties [8]. However, especially for expensive or invasive diagnostic procedures, referrals are requested from a GP or a specialist in ambulatory care. Normally, the GP or specialist in primary care decides on the indication and performance of MRI or CT scan and then refers the patients directly to the radiologist, who works in a community-based practice. The indication for an intracardiac catheter is set by a cardiologist at the hospital or in a community-based practice in most cases. For routine diagnostic or follow up, but not for emergency case, a referral from a GP is formally required.

This prospective observational study examined referrals for test ordering with respect to the physicians' perceived usefulness of high-cost diagnostic imaging for diagnosis and treatment in general practice. Our aim was to identify clinical conditions where GPs and specialists in ambulatory care perceived limited relevance of the tests so that targeted strategies could be planned to support medical decision making in these conditions. Because of the impact on the financial resources the study focused on high-cost imaging, including MRI scan, CT-scan and intracardiac catheter.

## Methods

### Design

The project was designed as a prospective observational study. 34 general practitioners of a medical network, one neurologist and one orthopaedic specialist in ambulatory care and one hospital of the region participated between January and March 2000. In this time period all referrals concerning MRI scan, CT-scan or intracardiac catheter from a total of 234 patients were documented by the physicians. The participating physicians were members of the Medical Quality Network Ried in the southern region of Hessia, Germany. The overall aim of this network is the implementation of continuous quality improvement in primary care. For instance, previous activities focused on the introduction of a patient-held medical record [9].

### Measures

The participating physicians were instructed to document each referral for MRI, CT-scan or intracardiac catheter. The documentation included reason for referral, clinical symptoms and previously established diagnoses of each patient.

The main outcome measure was the assessment by the physician if the result of the diagnostic procedure represents an important contribution to the diagnostic process. This could be a confirmation or negotiation of the estimated diagnoses for providing optimal treatment respectively to reassure that there is no dangerous health problem. To clarify this concept, a workshop with the participating physicians at the start of the study was done, in which the physicians were instructed to use this operationalization. The statement could be given as "useful" or "not useful" after receiving the test result. The estimation of the usefulness was usually made on the same day, when the physician received the referral letter. In Germany, the specialist who performed the diagnostics is obliged to send such a letter to the referring physician where the test results are listed in detail. For every documentation of the referral process and estimation of its usefulness, the physicians received a small financial incentive.

### Analysis

Every reason for a diagnostic referral was documented in a free text. The clinical symptoms were clustered into six different subgroups after analysis of the whole spectrum of referrals: cervical and lumbar spine, internal diseases (without cardiac problems), nervous system, musculoskeletal system, cardiac system and others. Baseline data were compared descriptively. The Chi-Square-Test was used for testing the relation between gender of patients and perceived usefulness statistically. The t-test was performed to calculate the relation between perceived usefulness (as independent variable) and age (as dependent variable). Statistical procedures and analysis of the data were done with SPSS 11.0.

## Results

Referrals for test ordering from 132 women and 100 men were documented (table 1). In sum 80 % of the diagnostic referrals of the women were assessed as useful, while 75 % were assessed as useful for the male patients. The Chi-Square-Test showed that this difference is not statistically significant (p = 0.796). There was no association between patient age and perceived usefulness of the test (t-test, p = 0.307).

**Table 1:** Characteristics of Patients: (in brackets: useful / not useful / no assessment)

**Sex**	**N**	**Age**	**CT**	**MRI**	**Cath.**	**All**	**useful in % (95% CI)**
Female	132	54.8 + 16.0 min 17; max 85	51	68	13	(106 / 23 / 3)	80,3 (72.7–86.2)
Male	100	56.0 + 16,6 min 13; max 85	44	28	28	(75 / 18 / 7)	75,0 (65.7–82.5)
No declaration	2	42 y, 51 y	1	1	0	(2 / 0 / 0)	100

Amount	234	55.3 + 16.2 min 13; max 85	96	97	41	(183 / 41 / 10)	78,2 (72.5–83.0)

Table 2 describes the perceived usefulness of tests in different clinical conditions. In cerebral diseases, MRI and CT-scans were perceived to be useful for diagnosis in apoplexia (100% resp. 83%, in sum 89%) and encephalitis disseminata (ED) (75%). A high amount of MRI was ordered by the neurologist to rule out a cerebral tumour by reason of persistent headache (perceived usefulness 89%). The usefulness was perceived to be low in unclear cerebral symptoms (suspected disease), mostly due to persistent dizziness, ataxia, tinnitus and other vague complaints (37% resp. 50%, in sum 40%).

**Table 2:** Performed Diagnostics: (in brackets: useful / not useful / no assessment)

**Area**	**GP / Specialist**	**Indication**	**CT**	**MRI**	useful in % (95% CI)
**Cerebral (n = 61)**	GP	Apoplexia (3)	3 (3 / 0 / 0)	0	100
	GP	Cerebral Tumour (3)	1 (1 / 0 / 0)	2 (2 / 0 / 0)	100
	GP	ED* (4)	1 (1 / 0 / 0)	3 (2 / 1 / 0)	75.0
	GP	Suspected Disease (11)	5 (2 / 2 / 1)	6 (2 / 3 / 1)	36.4
	Neuro.	Apoplexia (6)	2 (1 / 1 / 0)	4 (4 / 0 / 0)	83.3
	Neuro.	Rule out tumour (27)	8 (6 / 2 / 0)	19 (18 / 1/ 0)	88.9
	Neuro.	Trauma / SVT** / Hydrozephalus (3)	1 (1 /0 / 0)	2 (2 /0 / 0)	100
	Neuro.	Suspected Disease (4)	1 (1 /0 / 0)	3 (1 /2 / 0)	50.0

Total		Amount (61)	22 (16 / 5 / 1)	39 (31 / 7 / 1)	77.0 (65.1–85.8)

**Vertebra (n = 71)**	GP	Cervical spine (12)	5 (2/3/0)	7 (3 /2 / 2)	41.6
	GP	Lumbar spine (26)	20 (17/2/1)	6 (5 /0 / 1)	88.5
	Orthop.	Cervical spine (8)	3 (3 /0 / 0)	5 (3 /2 / 0)	75.0
	Orthop.	Lumbar spine (9)	6 (5 /1 / 0)	3 (2 /1 / 0)	77.8
	Neuro.	Cervical spine (8)	1 (1 /0 / 0)	7 (6 /1 / 0)	87.5
	Neuro.	Lumbar spine (8)	2 (2 /0 / 0)	6 (6 /0 / 0)	100

Total		Amount (71)	37 (30 / 6 / 1)	34 (25 / 6 / 3)	77.5 (66.5–85.6)

**Musculoskeletal Symptoms (n = 18)**	GP	Knee (10)	0	10 (9 / 1 / 0)	90.0
	GP	Shoulder (3)	0	3 (3 / 0 / 0)	100
	GP	Elbow (1)	0	1 (1 / 0 / 0)	100
	Orthop.	Knee (1)	1 (1 / 0 / 0)	0	100
	Orthop.	Shoulder (2)	0	2 (2 / 0 / 0)	100
	Orthop.	Upper ankle (1)	0	1 (1 / 0 / 0)	100

Total		Amount (18)	1 (1 / 0 / 0)	17 (16 / 1 / 0)	94.4 (74.2–99.0)

**Area**	**Indication**		**CT**	**MRI**	useful in % (95% CI)

**Internal (n = 27)**	Abdomina l complaints (15)		15 (10 / 5 / 0)	0	66.7
	Pulmonary (7)		6 (5 / 0 / 1)	1 (1 / 0 / 0)	85.7
	Urogenital (2)		2 (2 / 0 / 0)	0	100
	Other tumours (3)		2 (1 / 1 / 0)	1 (1 / 0 / 0)	66.7

Total	Amount (27)		25 (18 / 6 / 1)	2 (2 / 0 / 0)	74.1 (55.3–86.8)

**Others (n = 4)**	Chron. Sinusitis		1 (1 / 0 / 0)	0	100
	Lymphhaemangioma axillae		0	1 (1 / 0 / 0)	100
	Pain after Appendectomy		1 (1 / 0 / 0)	0	100
	Thoracic pain of unknown origin		1 (1 / 0 / 0)	0	100

Total	Amount(4)		3 (3 / 0 / 0)	1 (1 / 0 / 0)	100

**Area**	**Indication**		**Intracardiac catheter**	**MRI**	**useful in % (95% CI)**

**Cardiac (n = 49)**	Coronary artery disease		48 (39 / 6 / 3)	0	81,3 (68.1–89.8)
	Cardiac effusion		0	1 (1 / 0 / 0)	100

* ED = Encephalitits disseminate; ** SVT = sinus vein thrombosis GP = general practitioner, Neuro. = neurologist, Orthop. = orthopaedic specialist

The usefulness of tests for cervical spine problems was less than 42% for the GPs, for the orthopaedic specialist 88% and for the neurologist 75% (in sum 64%). In opposite to that, both the GPs and specialists perceived MRI and CT-scan as useful for lumbar spine problems in about 88–100%. Problems with the knees, shoulder, elbows and foot ankles had the best result regarding usefulness of test ordering (in sum 94 %).

In cases of abdominal complaints, where also cancer was suspected as a reason, 67% of the diagnostic referrals were perceived as useful. "Unexplained abdominal symptom" was the most frequent reason for ordering an abdominal CT-scan (seven out of 15 cases). The usefulness of tests for pulmonary diseases, mostly for suspected carcinoma, was higher, but the absolute number of cases was limited. The group of "other tumours" included two cases of breast cancer and one case of oesophageal cancer. In cases of cardiac problems most tests referred to suspected coronary artery disease (CAD), using intracardiac catheters. One MRI was done because of a suspected pericardial effusion. In sum, the cardiological diagnostics were perceived as useful in 82%.

## Discussion

The aim of our study was to examine to what extent MRI, CT-Scan or intracardial catheter were perceived as useful for making diagnoses and treatment decisions from the point of view of the physicians. The usefulness was most limited in problems related to cervical spine, unclear cerebral symptoms and abdominal pain.

In general, there is a limited usefulness of MRI and CT-scan for diagnostics in cervical spine problems [10,11]. The challenge in general practice concerning the management of patients with cervical spine problems is the already known psychological co-morbidity in this disease [12]. Due to the psychological strain of the patients related to a suspected herniated disc or nerve inflammation, there is a high pressure to clarify the reason of pain or to rule out a severe disease. As the test result is often negative whilst the pain is persisting, physicians and patients could be both disappointed, thus leading to low perceived usefulness of high-cost imaging.

In opposite to that, the diagnostic procedures in low back pain were perceived as useful in most cases. However, clinical research showed little correlation between morphology and complaints in low back pain [13]. The value for physicians in these cases could be the possibility to reassure the patient that there is no "dangerous problem". The optimistic estimation could reflect the satisfaction with care and with the course of the disease, which is normally inherent benign as the moderate lumbago is often self limiting.

The comparatively low usefulness of CT-scans in abdominal complaints may express the complexity of diagnostics in these cases. Many patients with unexplained abdominal symptoms show a high psychological co-morbidity [14], which could influence the doctor-patient-interaction and lead to a higher rate of diagnostic tests [6]. The same may be the case in unexplained neurological symptoms, which are often related with anxiety or depressive disorder [15]. Even if a perceived usefulness of about 64% could be seen as high, it should be mentioned that MRI or CT as most expensive tests are often done at the end of diagnostic procedures. The value of these tests should also be considered critically as they may induce unnecessary somatisation and medicalisation of the patients. To summarize, these results underline the difficulties in clinical diagnostics, particularly regarding unexplained complaints, which are frequent causes for consultation [16]. The problem behind is the high psychological co-morbidity, which is associated with prolonged illness behaviour and provocation of high usage of diagnostic procedures with an additional risk to harm the patients [17,18].

The results concerning the tests for cardiac diseases and problems with the musculoskeletal seem to be more informative. Intracardiac catheter, MRI or CT-scan led in most of these cases to an effective medical decision making, so it was regarded as helpful and appropriate by the referring GP.

A limitation of our study was that we investigated a subjective estimation of the physicians for the usefulness of the diagnostic referral but not the appropriateness judged by an external observer. For example, in the field of cardiology the managing of coronary artery disease in Germany has been criticised [19]. This indicates that the appropriateness for cardiological diagnostics could be weak despite the optimistic estimation in our study. Another limitation is that we had no total control on the response rate. But as the physicians received financial incentives for every documentation we assume that the response rate was quite high. The participating physicans are members of a medical quality network with a high motivation for participating in quality improvement projects. It must be questioned if the results are representative or even if there could be an overestimation of the usefulness regarding test ordering. The number of clinical conditions was large, consequently the subdivision into six categories led to heterogeneous groups with comparatively high confidence intervals. Further research should confirm the results of this explorative study.

Nevertheless, our study suggests that quality improvement should focus on patients with unexplained complaints to avoid expensive, unnecessary or dangerous diagnostic investigations. A starting point for dealing with these problems could be an analysis together with the network of physicians and a subsequent implementation of evidence based guidelines, accompanied by training in risk communication with difficult patients. This implementation of change should be done in a multifaceted strategy using guidelines, feedback and social interaction [20,21].

## Conclusion

The perceived usefulness of high-cost diagnostic imaging was lower in unexplained complaints than in specific diseases. Interventions to improve the effectiveness and efficiency of test ordering should focus on clinical decision making in conditions where GPS perceived low usefulness. Further research is necessary to identify patient factors underlying unexplained symptoms and to find methods to improve decision making regarding test ordering.

## Competing interests

The author(s) declare that they have no competing interests.

## Authors' contributions

AS performed the statistical analysis and wrote the manuscript. TR supported in writing the manuscript. MW supported in statistics and writing. JS designed and supervised the study. All authors read and approved the final manuscript.

## Pre-publication history

The pre-publication history for this paper can be accessed here:


